# Pediatric Manubriosternal Dislocation: A Case Report and Review of Literature

**DOI:** 10.7759/cureus.14163

**Published:** 2021-03-28

**Authors:** Shannon Yancovich, Andrew Wahba

**Affiliations:** 1 Pediatrics, McGovern Medical School, University of Texas Health Science Center, Houston, USA

**Keywords:** manubriosternal dislocation, trauma, chest pain

## Abstract

Pediatric sternal dislocation is an extremely rare event, with less than 15 cases reported in the literature. We report the case of a traumatic sternal segment dislocation in an 8-year-old male that was caused by an unsuccessful backflip resulting in a direct force to the chest. The diagnosis was made by chest computed tomography. Open reduction and internal fixation (ORIF) were performed with a good outcome noted at follow-up. In addition to a thorough description of this case, we have included an organized review of existing literature with an aim to establish trends in presentation, clinical course, and outcomes among sternal dislocation in the pediatric population.

## Introduction

The sternum consists of the manubrium at the most cranial end, followed by the four segments of the body (called sternebrae), then finally the xiphoid process at the most distal end. In early childhood, the components of the sternum are joined by synchondrosis (primary cartilaginous joint). The segments of the sternum begin synostosis (fusion) in a craniocaudal direction in a process that typically starts at 7 years of age [1,2]. Synostosis is typically completed by 25 years of age, although there is great variation in the timing of synostosis, with a subset of individuals having unfused or only partially fused sternum into adulthood [1]. Pediatric sternal dislocation is a rare complication with very limited data reported in the literature [3]. Most occurrences are due to trauma, from either direct or indirect forces on the anterior chest wall, although other causes such as osteomyelitis and osteonecrosis have been previously described [4-6]. The absence of fusion is likely the reason why pediatric populations report higher instances of dislocation versus sternal fracture. Sternal fracture (with or without dislocation of the sternum) is more commonly reported in adult populations, given the majority of the adult population would have fused sternal bodies.

Two types of sternal dislocation have been described based on the relation to the manubrium on the sagittal axis. Type one is posterior dislocation of the sternal body caused by high energy direct trauma. Type two is dislocation of the manubrium caused by indirect flexion or rotational injury. Dislocation of the first segment of the sternal body (the manubriosternal joint, or MSJ) is the most commonly reported site followed by second and third segments. Dislocation at the MSJ is likely more prevalent given that the manubrium on the superior aspect of the joint is firmly attached to the clavicles and adjacent ribs. The first sternebrae and its inferior elastic chest wall are more compliant than the manubrium, so when challenged with a significant displacing force, the first sternebrae would be compressed inward while the manubrium would remain relatively static, creating a shearing force that could result in dislocation.

We report the case of a traumatic sternal segment dislocation in an 8-year-old male that was caused by an unsuccessful backflip resulting in a direct force to the chest. We review his presenting symptoms, imaging features, treatment, and outcome as well as the relevant literature of this rare entity in the pediatric population.

## Case presentation

An 8-year-old previously healthy male presented to the emergency department with complaints of mild dull anterior chest wall pain after sustaining an injury to his chest one week prior. The injury occurred when he was attempting to do a backflip while in an inflatable bounce house and landed incorrectly, resulting in his flexed lower extremities causing a direct blow to his anterior chest wall. He endorsed brief chest pain and breathlessness following the event but was able to resume playing minutes after the injury without any respiratory distress. Over the next several days, the patient noted intermittent chest pain. It was non-radiating, and not accompanied by shortness of breath or dysphagia. The pain was worse with exertion and positions that resulted in the extension of his back and shoulders. His mother noted his posture gradually became “hunched” and that he had a predilection for holding his arms midline in front of his body. Due to the persistence of the pain and worsening posture, they sought medical care seven days after the injury. Of note, he denied any recent history of fever, cough, dyspnea, easy bleeding or bruising, and unintended weight loss.

On physical examination, the oral temperature was 98°F, heart rate 98 beats per minute, blood pressure 112/70 mmHg, respiratory rate 18 times per minute, and oxygen saturation was 100% on room air. Weight was 56.4 kg with a body mass index (BMI) of 19.7 kg/m^2^. There was no palpable deformity but he endorsed mild tenderness of the sternum. The patient moved all extremities appropriately with a full range of motion but did endorse pain with thoracic movement. No signs of marked stiffness or joint deformities were evident in other joint examinations. Lungs were clear to auscultation bilaterally with good air movement. Upon auscultation of the heart no murmurs, rubs, or gallops were appreciated. The abdomen was soft, non-distended, non-tender with no hepatosplenomegaly. On a neurological exam, he was awake and followed commands, and was able to move all extremities with intact cranial nerves, sensations, and reflexes. No jaundice, oral ulcers, thrush, lymphadenopathy, petechiae, ecchymosis, or signs of bleeding were present.

**Figure 1:**
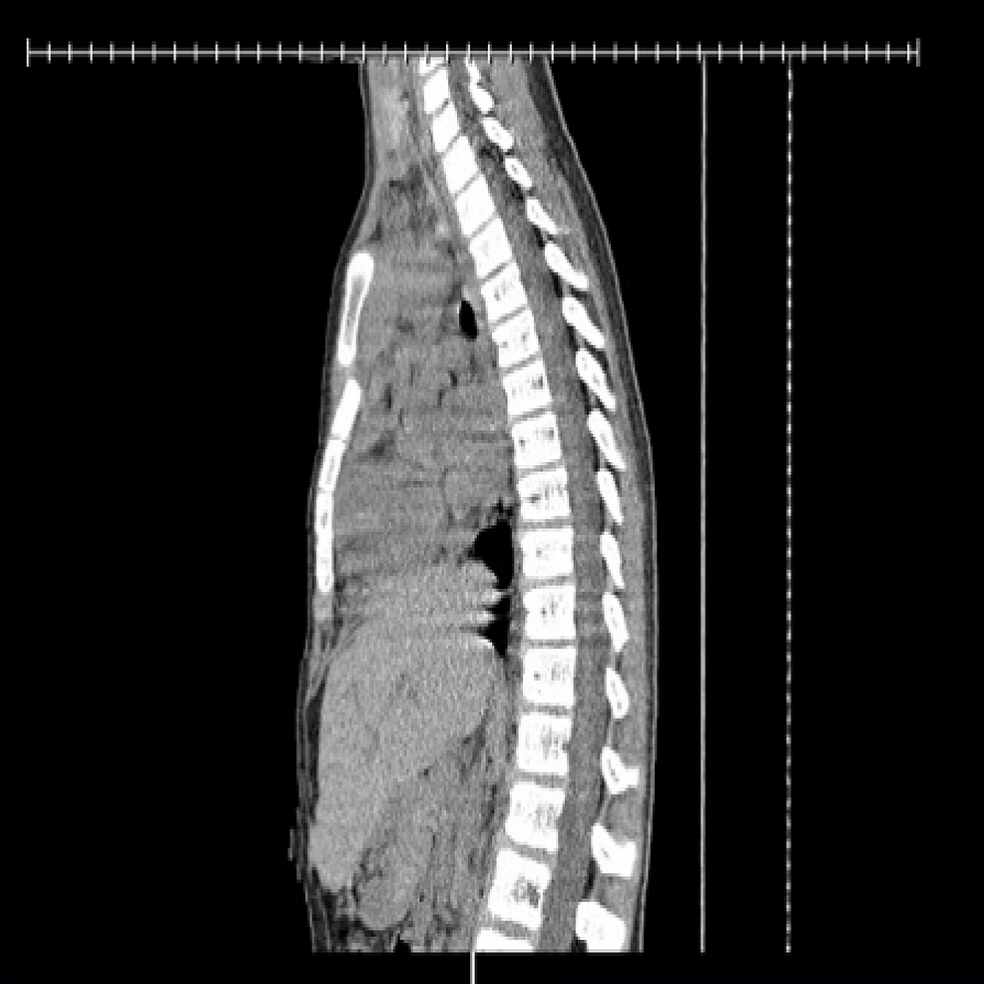
CT scan of the chest Computed tomography scan of the chest, lateral view demonstrating the posterior dislocation of the upper sternal body with respect to the manubrium. The dislocated upper sternal segment is approximately a whole sternal thickness posterior to the manubrium.

**Figure 2:**
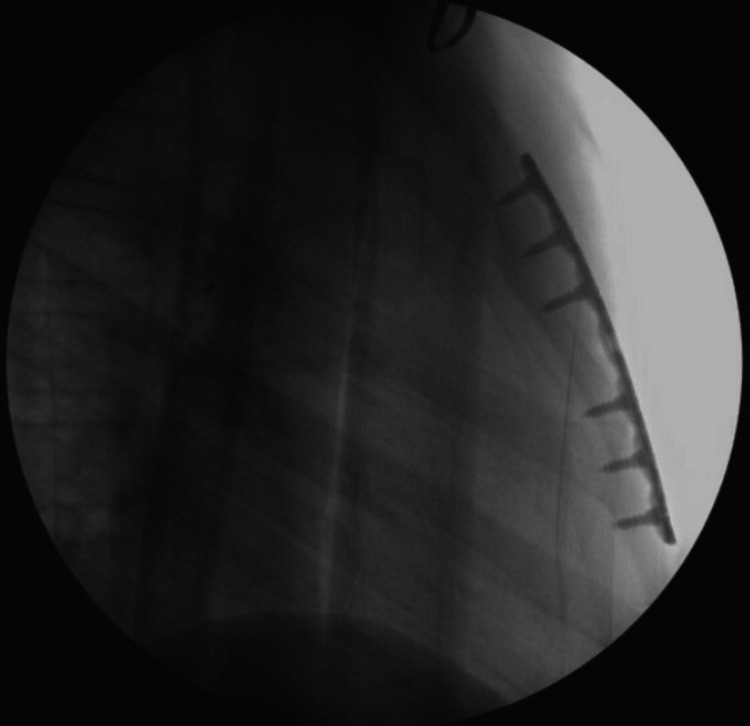
Intraoperative fluoroscopy Intraoperative fluoroscopy demonstrating surgical correction of the dislocated sternebrae with the placement of a metal plate and six sternal screws with the achievement of appropriate alignment.

Complete blood count and comprehensive metabolic profile were within normal limits. Troponin was <0.02 nanograms/milliliter and Electrocardiography (EKG) showed normal sinus rhythm with a PR interval of 130 milliseconds (ms), QRS 80 ms, and the corrected QT interval (QTC) 422 ms. CT chest (Figure 1) revealed posterior dislocation of the upper sternal body by one cortex width with respect to the manubrium, a possible retrosternal hematoma, and mild bibasilar subsegmental atelectasis. He was admitted to the pediatric surgery service and underwent an open reduction and internal fixation (ORIF) with metal plating of the manubriosternal junction on post-injury day 8. Intraoperative fluoroscopy confirmed placement and good alignment of the repair (Figure 2). The patient noted improvement in his chest pain in the immediate postoperative period. At two-week post-operative follow-up, he was noted to have appropriate healing of the surgical site and stability of the sternum. He had no complaints of chest pain while on light activity restriction with the return to full activity at six-week post-operative.

## Discussion

Chest pain in the pediatrics population can be categorized into two broad subsets non-traumatic and traumatic. Non-traumatic can be due to a wide variety of etiologies including cardiac, pulmonary, esophageal, musculoskeletal, psychiatric, or neuropathic. Of the non-traumatic causes, it is estimated that only about 1%-6% are life-threatening conditions [7,8]. Pediatric blunt thoracic trauma, as one might expect, can have more daunting consequences with retrospective studies estimating up to 4%-14% resulting in death [7,8]. The cartilaginous and bony structures of the pediatric thorax are distinct from adults in that they are significantly more elastic and compressible. As a result, the pliable chest has the potential to absorb higher amounts of kinetic energy which can successively be transmitted to intrathoracic structures. This concept is critical to consider because it is not uncommon for intrathoracic injuries to occur in the pediatric population despite an otherwise well-appearing exterior chest wall. Upon literature review, multiple cases reported inward rotation of the dislocated sternal segment from the plane of the sternum. In 58% of the cases, the dislocated segment was rotated 90 degrees and 21% was noted to be roughly 45 degrees rotated. In two patients, the segments progressively rotated with time [5,9]. Wada et al. noted the sternal segments were marginally displaced (~45 degrees) initially after the dislocation, no intervention was performed and the segment continued to rotate until it assumed a more stable orientation of 90 degrees from the sternum at the two weeks' follow up [9]. Of the cases reviewed, eight mentioned a palpable chest wall deformity, either initially or later in the course of the injury. Of these cases, one was noted to have an audible click when palpated [10]. Kusuba et al. noted that the dislocated (sternebrae 2) segment was so unstable that the bony deformity visibly moved with respiration [11]. As one might expect, the literature reviewed supported that the larger the degree of rotation, the more likely there was a palpable deformity on a physical exam. Of those patients who were noted to have an initial palpable deformity on physical exam, 85% were found to be associated with a 90-degree rotation. Conversely, only one patient with a 45-degree rotation of the sternal segment was noted to also have a palpable deformity, although soft tissue swelling was seen in some of the patients in this subset.

To date there is no estimated prevalence of sternal dislocation in the pediatric population, likely owing to the fact that it is such a rarity. A MEDLINE search was conducted for queries including “Manubriosternal dislocation”, “Sternal dislocation”, “Sternal fracture” and “Thoracic trauma”. Relevant papers were selected for the literature review. Twelve cases of sternal dislocation have been previously reported in the pediatric population [3-5,8-14]. In most cases, sternal dislocation was caused by either direct force (blunt trauma to the anterior chest wall) or indirect forces. Reports of indirect forces resulting in sternal dislocation include rotational forces, hyperflexion injury transmitted through the clavicles, hyperextensions of the thorax, and one documented case that occurred after occasional coughing. Thus far, only two cases of sternal dislocation have been documented that were not caused by direct or indirect forces. One was a 10-year-old male with staphylococcus aureus osteomyelitis of the sternum who suffered sternal dislocation one week after intravenous antibiotic therapy [5]. Another was a 5-year-old Caucasian male with sternal osteonecrosis (avascular necrosis [AVN]) of unknown etiology [6]. The mean age for sternal dislocation was 6 years old (ages ranged from 18 months to 14 years). 63% of cases occurred in ages less than 7 years and 81% of the cases were males. Also, 50% were found to be caused by direct force, 38% caused by indirect force, and the remaining 12% caused by other etiologies (osteomyelitis, and AVN). Of the cases reviewed 75% involved the MSJ (first sternebrae). Only one case had associated sternal fracture [4]. The mean time to seek medical care after symptom onset was 4.9 days. All patients who sustained sternal dislocations reported anterior chest wall pain. Most patients reported the pain to be intermittent in nature, with 38% reported increased pain with increased intrathoracic pressure (such as Valsalva and coughing) and 23% reported worsening pain with physical exertion and thoracic movements.

Although the majority of patients had chest pain as their only complaint, 15% also reported associated symptoms of dyspnea and/ or dysphagia, prompting concerns for the integrity of the surrounding tissues. In the adult population, there have been accounts of pulmonary contusion associated with sternal fractures but thus far no pulmonary pathology has been definitively reported as a result of sternal dislocation in the pediatric population. Kusaba et al. did report a 4-year-old male with a direct blow to the chest with pain who developed a cough on day 9 after injury which exacerbated his chest pain [11]. In this case report, the cough was thought to be due to a suspected “cold” but also hypothesized that it could have been secondary to pleural irritation caused by the dislocated segment given his lack of other infectious symptoms such as congestion and fever.

Given the location of the esophagus in the posterior mediastinum, concomitant esophageal involvement seems to be a less potential outcome. Two cases in the pediatric population, however, did have symptoms of dysphagia one of which was evaluated with a barium swallow study, but results were unremarkable [6,13]. There have also been documented cases of adults with sternal dislocation and or fracture who sustained a concurrent spinal injury. Such occurrences have yet to be reported in the pediatric population, again possibly owing to the elastic nature of the pediatric chest wall [15].

When considering the structure and location of the sternum it’s rather surprising that no pediatric cases in our review were found to be associated with intrathoracic trauma. Although none have been documented thus far, there is literature to at least support the potential for damage to proximal structures (primarily, the right ventricle and the lungs). The sternum is directly anterior to the heart, with the right ventricle of the heart comprising the majority of its sternal border and the pulmonic valve in its vicinity. The aortic arch is approximately at the level of T4, the second rib, and the MSJ. The proximity of the right ventricle, aorta, and pulmonic valve to the sternum begs the question of a possible secondary injury to these structures in the event of a sternal dislocation. In our review of literature, EKG was performed in 23% of the cases and CT of the chest in 30% to further evaluate for possible cardiothoracic involvement however all of which were normal. Although there are no reports of cardiothoracic injury associated with sternal dislocation in the pediatric population, there have been cases of adult sternal fractures that were reported to have concurrent injuries to the heart including myocardial contusion, myocardial laceration, and aortic injury [12]. Robicsek et al. described the case of a 16-year-old male who developed severe extrinsic pulmonary stenosis caused by large callus formation at the site of previous transverse sternotomy for teratoma removal [16]. While it is not the same etiology, the location of a possible sternal callus from sternal dislocation would be geographically similar to that of a post-sternotomy callus, thus sparking the question of the potential involvement of the pulmonic valve in the event of a sternal dislocation. Interestingly enough, sternal dislocation in mice has been documented to cause histological changes in the right ventricle. Adissu et al. noted 22 of the 51 mice with sternal segment dislocation (with particularly severe displacement and/or large callus formation) demonstrated adjacent right ventricle epicardial fibrosis [17]. Such a histologic pattern is known to have clinical correlations of conduction slowing and arrhythmia. Similar studies have not been evaluated in the pediatric population that suffered sternal dislocation however such effects certainly seem to be plausible given the geographic location of these entities. These reports emphasize the consideration of a cardiac workup despite the lack of documented occurrences of cardiac dysfunction in the pediatric population.

Most diagnoses of the literature reviewed were made from lateral chest radiographs (many anterior-posterior or PA views were read as normal), one case was initially diagnosed by ultrasound, then later confirmed by CT. In total, one-fourth of the cases had chest CT performed to further assess the dislocation and to look for any signs of concomitant intrathoracic damage which no cases found. Over the years there have been two main approaches to the management of sternal dislocation: reduction or observation, the exception to this being one patient with AVN whose affected sternebrae was excised. The reduction can be performed in either a closed (via manual extension maneuver) or open (via ORIF) manner. Of the articles reviewed, 40% was managed with open reduction, 40% with observation, one patient who was initially observed ultimately required ORIF due to failure under observation, one patient with excision, and one with observation following intravenous antibiotic therapy. In patients who were managed with open reduction, all surgeons used metal plates and screws except two (one of which used K wires and another used bio adorable pins). Of the eight studies that were reassessed for bone remodeling after the intervention, the earliest that partial remodeling was noted was three months post-injury in a patient that was managed conservatively with observation [5]. The earliest completed remodeling was noted at seven months post-injury in a patient that was managed conservatively with observation [14]. Of the five pediatric cases that noted completed remodeling, the average time was 42 months post-injury [4,9].

## Conclusions

Manubriosternal dislocation should be considered in the differential diagnosis of pediatric patients with chest trauma. There are two types of pediatric sternal dislocation depending on the mechanism of action and the relation to the manubrium. Most patients are asymptomatic or complain of mild chest pain with fewer cases reported dysphagia or dyspnea. Management is either by observation or open reduction with favorable outcomes in all patients. This case advances the literature by providing extensive review with an aim to establish trends in presentation, clinical course, and management.
